# Reduced binding activity of vaccine serum to omicron receptor-binding domain

**DOI:** 10.3389/fimmu.2022.960195

**Published:** 2022-07-28

**Authors:** Mingzhi Li, Shiqi Weng, Quansheng Wang, Zibing Yang, Xiaoling Wang, Yanjun Yin, Qiuxiang Zhou, Lirong Zhang, Feifei Tao, Yihan Li, Mengle Jia, Lingdi Yang, Xiu Xin, Hanguang Li, Lumei Kang, Yu Wang, Ting Wang, Sha Li, Lingbao Kong

**Affiliations:** ^1^ Nanchang City Key Laboratory of Animal Virus and Genetic Engineering, Institute of Pathogenic Microorganism, College of Bioscience and Engineering, Jiangxi Agricultural University, Nanchang, China; ^2^ GMU-GIBH Joint School of Life Sciences, Guangzhou Medical University, Guangzhou, China; ^3^ Department of Clinical Laboratory, Jiangxi Provincial Children’s Hospital, Nanchang, China; ^4^ Department of Clinical Laboratory, The Affiliated Hospital of Jiangxi Agricultural University, Nanchang, China; ^5^ College of Animal Science and Technology, Jiangxi Agricultural University, Nanchang, China; ^6^ Center for Laboratory Animal Science, Nanchang University, Nanchang, China

**Keywords:** COVID-19, omicron, spike protein, RBD, binding activity

## Abstract

Coronavirus disease 2019 (COVID-19) vaccination regimens contribute to limiting the spread of severe acute respiratory syndrome Coronavirus-2 (SARS-CoV-2). However, the emergence and rapid transmission of the SARS-CoV-2 variant Omicron raise a concern about the efficacy of the current vaccination strategy. Here, we expressed monomeric and dimeric receptor-binding domains (RBDs) of the spike protein of prototype SARS-CoV-2 and Omicron variant in *E. coli* and investigated the reactivity of anti-sera from Chinese subjects immunized with SARS-CoV-2 vaccines to these recombinant RBDs. In 106 human blood samples collected from 91 participants from Jiangxi, China, 26 sera were identified to be positive for SARS-CoV-2 spike protein antibodies by lateral flow dipstick (LFD) assays, which were enriched in the ones collected from day 7 to 1 month post-boost (87.0%) compared to those harvested within 1 week post-boost (23.8%) (*P* < 0.0001). A higher positive ratio was observed in the child group (40.8%) than adults (13.6%) (*P* = 0.0073). ELISA results showed that the binding activity of anti-SARS-CoV-2 antibody-positive sera to Omicron RBDs dropped by 1.48- to 2.07-fold compared to its homogeneous recombinant RBDs. Thus, our data indicate that current SARS-CoV-2 vaccines provide restricted humoral protection against the Omicron variant.

## Introduction

COVID-19 is a worldwide pandemic caused by SARS-CoV-2. Although multiple measures have been adopted, COVID-19 is still rife amid the world and poses a threat to social, mental, and economic wellbeing (1, 2). The newly evolved Omicron mutant spread quickly within highly vaccinated populations. Viral sequence analysis reveals that as the most heavily mutated variant, Omicron harbors 15 mutations in its spike RBD region. Considering that the RBD domain of the SARS-CoV-2 spike protein mediates the viral entry, thus contributing to viral infection and transmission, a primary concern arises about the effectiveness of the current vaccine regimen against this viral variant (3). To this end, we expressed the RBD monomer and dimer of Omicron spike protein and examined the cross-reactivity of anti-sera from subjects immunized with prototype SARS-CoV-2 vaccines (either inactivated vaccines or RBD dimer subunit vaccines) to Omicron RBDs. Our data showed that vaccine-immunized sera displayed reduced binding activity to Omicron RBDs, implying the low efficacy of the prototype SARS-CoV-2 vaccine to protect against the Omicron variant.

## Methods

### Materials

Ninety-three human serum samples from 78 individuals immunized with prototype SARS-CoV-2 vaccines including inactivated whole-virus vaccines Sinopharm BBIBP-CorV and Sinovac CoronaVac (n = 62), Sinopharm BBIBP-CorV (n = 16), Sinovac CoronaVac (n = 14), or RBD dimer-based subunit vaccine Zhifei ZF2001 (n = 1) were obtained from the Affiliated Hospital of Jiangxi Agricultural University and Jiangxi Children’s Hospital (**Supplementary Table 1**). Thirteen unimmunized serum samples from Jiangxi Children’s Hospital served as negative controls (**Supplementary Table 1**). All studies involving human sera were performed under the standard of the Jiangxi Agriculture University Ethical Committee. SARS-CoV-2 Antibody Detection Kits (Cat: W19501110, 2020340177, and Y5021010552A) were obtained from Wondfo, Innovita, and Vazyme of China, respectively. Antibodies against His-tag (RIID: AB_11,232,599), actin (RIID: AB_2,687,938), and HRP-labeled goat anti-human (Cat: SA00001-17) were purchased from Proteintech in USA. HEK293 cell-expressed RBDs of prototype SARS-CoV-2 (Cat: CSB-DP7031) and Omicron variant (Cat: 40592-V08H121) were obtained from CUSABIO and Sino Biological in China, respectively.

### Protein expression and purification

The coding sequence for spike RBD of Omicron strain B.1.1.529 (GenBank: PRJNA784547) was used for prokaryotic expression. The Omicron RBD dimer was synthesized in a tandem repeat form of the RBD monomer separated by their own flexible terminal residues (2). The corresponding sequence of the Omicron spike RBD monomer was amplified with the synthetic dimer sequence as the template. A similar strategy was used to amplify the monomeric and dimeric RBDs of prototype SARS-CoV-2 (GenBank: YP_009724390). All RBD DNA segments were cloned into the pET-28a plasmid for expression in *E. coli*. After induction at 18°C for 8 h in the presence of IPTG (0.5 or 1 mM), cells were collected to examine the recombinant protein expressions. All RBDs were further purified by the NI-NTA column followed by renaturation using dialysis and then concentration with Amicon^®^ Ultra-15 (10 or 30K) (4, 5).

### Immunoblotting

Immunoblotting was performed as described previously (6). Briefly, after electrophoresis on an SDS-PAGE gel, separated proteins were transferred to polyvinylidene difluoride membranes (Millipore). The membranes were blocked with 10% skimmed milk and then incubated with an antibody specifically targeting His-tag at 4°. Finally, the proteins were visualized with Clarity ECL immunoblotting substrate (Bio-Rad).

### LFD and ELISA

To detect SARS-CoV-2-specific antibodies in vaccine-immunized human sera, LFD assays were performed using SARS-CoV-2 Antibody Detection Kits according to the manufacturer’s instructions. ELISA was performed as described previously (4, 7). Briefly, plates were precoated with the recombinant RBDs (100 ng/well) at 4° overnight in 0.05 M carbonate-bicarbonate buffer. After blocking with 5% skim milk, human sera were diluted and added to each well. Goat anti-human IgG-HRP antibodies were then added. Plates were finally developed with TMB substrate. Commercial RBDs expressed in HEK293 cells were used to evaluate the quality of lab-made recombinant RBDs expressed in *E. coli*. To exclude the interference of His-tag reactivity, the anti-His antibody was also used in ELISA assays (**Supplementary Figure 2**). Optical density was measured at a wavelength of 450 nm using a plate reader (Tecan, Infinite M200 Pro).

### Statistical analysis

Student’s t-test and chi-square test were adopted to compare the intergroup differences using GraphPad Prism 8.0 software. *P* < 0.05 was considered statistical significance.

## Results

### Expression of monomeric and dimeric RBDs

**Figure 1:**
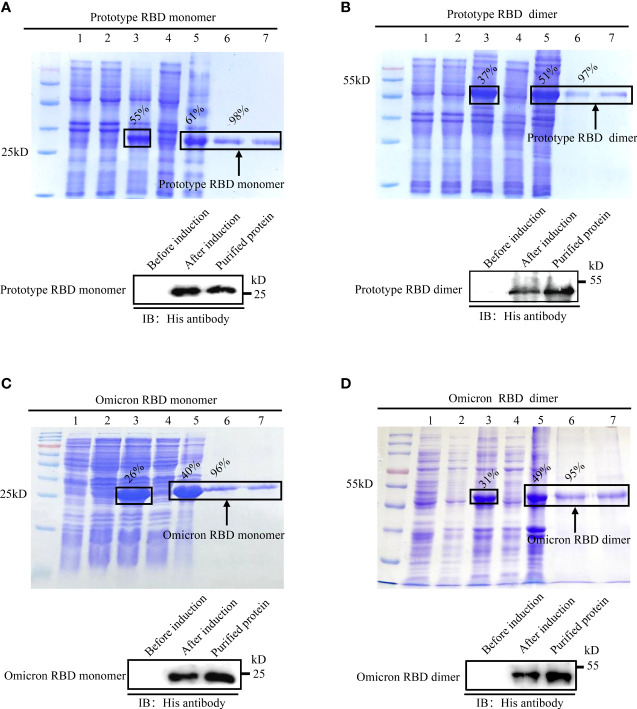
Expression, purification, and identification of recombinant spike RBDs. Recombinant pET-28a vectors expressing either monomeric or dimeric spike RBDs for prototype SARS-CoV-2 **(A, B)** and Omicron variant **(C, D)** were used to express the recombinant proteins in *E. coli.* The expressions and purities of RBDs were examined by SDS-PAGE (A-D, top) or immunoblotting (**A–D**, bottom). **(A-D)** Top: M: protein marker; lane 1: empty vector; lane 2: un-induced sample; lanes 3–5: IPTG induced whole-cell lysate (lane 3); cellular supernatant (lane 4); inclusion body (lane 5); lanes 6–7 (A-D): purified monomeric **(A, C)** or dimeric **(B, D)** RBDs in eluted buffer with 250 mM imidazole. **(A-D)** Bottom: identification of spike RBDs by immunoblotting with anti-His tag antibody.

Upon IPTG induction, the RBD monomer and dimer of the SARS-CoV-2 prototype and Omicron variant were expressed in *E. coli*, all of which dominated in the cellular inclusion bodies, accounting for 26%–55% of total protein mass (**Figures 1A**–**D**, top). After purification with Ni-NTA columns followed by the separation by electrophoresis on SDS-PAGE gel, intensive bands were detected for monomer and dimer RBD proteins in a buffer with 250 mM of imidazole. The purities of the recombinant RBD proteins were no less than 95% (**Figures 1A**–**D**, top). To confirm the identities of *E. coli*-derived recombinant proteins, we performed immunoblotting assays with an anti-His tag antibody. Expected bands were detected for all recombinant proteins in IPTG-induced lysate or purified samples, but not in un-induced ones, demonstrating the successful expression and purification of *E.coli*-expressed recombinant RBD proteins (**Figures 1A**–**D**, bottom).

### Cross-reactivity of prototype SARS-CoV2 vaccine-immunized sera against Omicron RBDs

**Figure 2:**
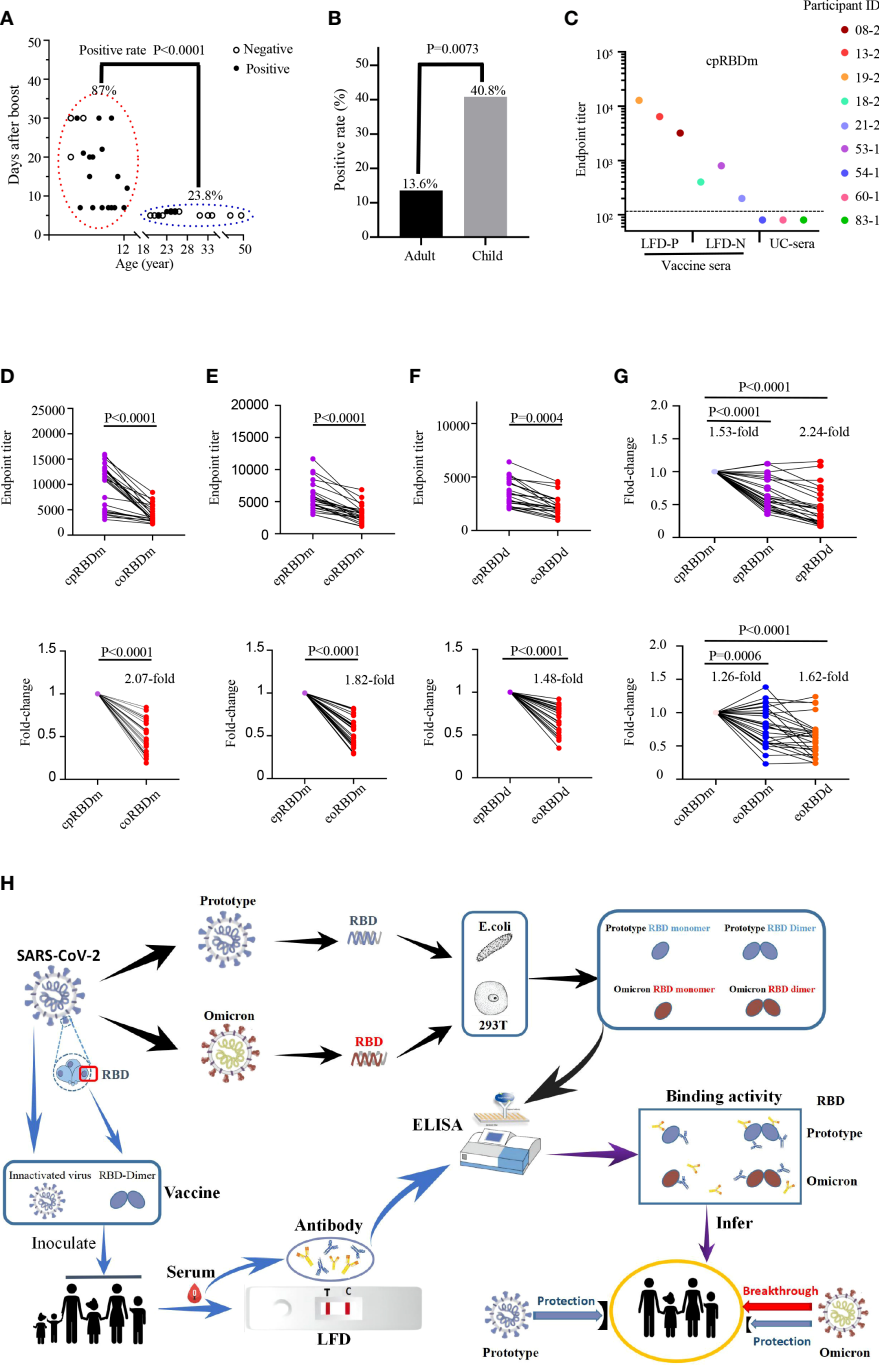
Reactivity of human sera with SARS-CoV-2 spike protein and recombinant RBDs. **(A, B)** Detection of anti-SARS-CoV-2 spike protein antibodies in human sera with LFD assays. **(A)** Examination of the contributions of age and sample collection time point to LFD-positive rates with the chi-square test. Children LFD-positive rate: (20 of 23, 86.9%); adult LFD-positive rate: (5 of 21, 23.8%). Child sera were collected at 7 days to 1 month after boost. Adult vaccine sera were collected within 1 week after boost. **(B)** Comparison of the LFD-positive rate of vaccine sera between adults (6 of 44, 13.6%) and children (20 of 49, 40.8%) with chi-square test. **(C)** Titration of SARS-CoV-2 RBD-specific antibodies in LFD-positive vaccine sera (participant IDs: 08-2, 13-2 and 19-2; LFD-P), LFD-negative vaccine sera (participant IDs: 18-2, 21-2 and 53-1; LFD-N), and unimmunized sera (participant IDs: 54-1, 60-1 and 83-1; UC-sera) by ELISA using the recombinant RBD monomer as coating proteins. The dashed line indicates the cutoff value. **(D-F)** Reactivity of 26 LFD-positive vaccine sera (further details in **Supplemental Figure 1A** and **Supplemental Table 1**) to commercial prototype and omicron RBD monomer **(D)**, *E. coli*-expressed prototype and omicron RBD monomer **(E)**, and *E. coli*-expressed prototype and omicron RBD dimer **(F)**. Top panels: absolute titers; bottom panels: fold change. cpRBDm/coRBDm: commercial prototype/omicron RBD monomer; epRBDm/eoRBDm: *E. coli*-expressed prototype/omicron RBD monomer; epRBDd/eoRBDd: *E. coli*-expressed prototype/omicron RBD dimer. **(G)** Fold change for comparison between commercial and lab-made RBDs. Prototype SARS-CoV-2 RBDs (top panel) and Omicron RBDs (bottom panel). Fold change is defined as mean fold change. Each dot represents a biological replicate, and the assays were performed three times **(A, C-G). (H)** Schematic diagram showing reduced binding activity of vaccine serum to Omicron RBD.

To assess the cross-reactivity of prototype SARS-CoV2 vaccine-immunized sera against Omicron RBDs, we first collected 106 blood samples based on availability. Among them, 44 samples were collected from 29 adults while the remains were from 62 children (**Supplementary Table 1**). LFD assays were conducted to identify vaccine sera with high-titer antibodies against prototype SARS-CoV-2 spike protein. Results revealed that 26 samples of 93 vaccine sera contained detectable antibodies specific to SARS-CoV-2 spike protein (**Supplementary Figure 1**). We next wondered the LFD-positive ratio of these samples by vaccine, age, sex, and post-immunization time. Therefore, a retrospective analysis was performed (**Supplementary Table 2**). The LFD-positive ratio for both BBIBP-CorV and CoronaVac immunization groups was 11 [17.7%] of 62 (adult: 6 [14.0%]/43, children: 5 [41.6%]/19). The LFD-positive ratio for the BBIBP-CorV immunization group was 7 [43.8%] of 16 (children). The LFD-positive ratio for the CoronaVac immunization group was 8 [57.1%] of 14 (children). The majority (20 [87.0%] of 23) of LFD-positive samples were the ones collected from day 7 to 1 month post-boost (BBIBP-CorV and CoronaVac immunization groups: (5 [83.3%] of 6); BBIBP-CorV immunization group: (7 [77.8%] of 9); CoronaVac immunization group: 8 [100%] of 8), different from those harvested within 1 week post-boost (5 [23.8%] of 21, BBIBP-CorV and CoronaVac immunization groups: 5 [23.8%] of 21; BBIBP-CorV immunization group: none; CoronaVac immunization group: none) (P < 0.0001, **Figure 2A**). A higher positive ratio was observed in the child group (20 [40.8%] of 49, BBIBP-CorV and CoronaVac immunization groups: 5 [26.3%] of 19; BBIBP-CorV immunization group: 7 [43.8%] of 16; CoronaVac immunization group: 8 [57.1%] of 14) than adult (6 [13.6%] of 44, BBIBP-CorV and CoronaVac immunization groups: 6 [13.6%] of 44; BBIBP-CorV immunization group: none; CoronaVac immunization group: none) (P = 0.0073, **Figure 2B**). The low LFD-positive ratio of vaccine sera is likely due to the limited sensitivity of the LFD assay. To test this possibility, we randomly chose three LFD-positive/-negative vaccine sera and three unimmunized sera to examine the quantities of antibodies targeting SARS-CoV-2 spike RBD. ELISA data showed that LFD-negative vaccine sera harbored small, but decent amounts of anti-SARS-CoV-2 spike RBD antibodies (**Figure 2C**).

To evaluate the binding activity of vaccine-immunized human sera to Omicron variants, LFD-positive sera were used. As shown in **Figures 2D**–**F**, although the tested sera cross-recognized Omicron spike RBDs, their reactivity magnitudes decreased by 1.48- to 2.07-fold compared to those of SARS-CoV-2 RBDs, which partially explained the rapid transmission of Omicron in the vaccinated regions. With the engagement of antibodies in human vaccine sera and RBDs as readout, general drops were observed for the *E.coli*-expressed prototype RBD monomer (1.53-fold, P < 0.0001), prototype RBD dimer (2.24-fold, P < 0.0001), Omicron monomer (1.26-fold, P = 0.0006), and dimer (1.62-fold, P < 0.0001) with the corresponding commercial ones as control (**Figure 2G**).

Of note, all recombinant RBD proteins carried a His-tag and recognized anti-His antibody with a reactivity corresponding to the quantity of its His-tag. In addition, recombinant RBD proteins recognized vaccine sera, but not unimmunized control sera (**Supplementary Figure 2**; **Figures 2C**–**F**). These data together suggested that the difference in vaccine serum reactivity to recombinant RBD proteins was not due to the anti-His antibody which could exist in vaccine sera.

## Discussion

Different from other SARS-CoV-2 variants, the Omicron strain occurs in the situation in which SARS-CoV2 vaccine immunization has been rolled out globally. Its fast spread in fully vaccinated countries such as the USA reveals that the existing vaccine provided limited protection. The World Health Organization (WHO) reported on February 15, 2022, that the Omicron variant had replaced the Delta variant as the main circulating strain worldwide. This study aimed to explore how the Omicron variant effectively evades the immune responses induced by heterogeneous SARS-CoV-2 vaccines. The CoV spike RBD is an attractive vaccine target. Its dimeric form fully exposes the dual receptor-binding motifs, thus significantly increasing neutralizing antibody (NAb) titers as compared to its conventional monomer (8). In this study, the Omicron spike RBD monomer and dimer were expressed and purified (**Figure 1**), serving as the antigens to evaluate the cross-protection of host immunity induced by prototype SARS-CoV-2 vaccines in the ELISA (**Figure 2**).

One hundred six blood samples from adults and children were collected and analyzed by LFD assays. The anti-SARS-CoV-2 spike protein antibody in vaccine sera collected at 5–6 months after the first boost dropped to below the detectable threshold, highlighting the necessity of the second boost (**Supplementary Figure 1**). Interestingly, vaccine sera at 7 days to 1 month after boost had a higher SARS-CoV-2 spike protein antibody titer than those within 1 week after boost (P < 0.0001, **Figure 2A**). Consistent with the finding, a recent report indicated that RBD antibody titers reached a plateau in 2 weeks or so after a boost, then dropped about fivefold within the following 2 weeks (9). These data suggest that boosted time affects the antibody titer of vaccine sera. Interestingly, a higher positive ratio was observed in the child group than in adults (P = 0.0073, **Figure 2B**), which could attribute to age besides the collection time points. Similarly, recent reports showed that immunity of CoronaVac for children seems better than that for adults (10, 11). It should be noted that LFD exhibited low sensitivity in detecting SARS-CoV-2 spike protein antibody of vaccine serum than ELISA (**Figure 2C**). Thus, the development of a more convenient and accurate CoV-2 antibody detection kit is warranted.

Coronavirus spike RBD is the key domain mediating the engagement between coronavirus and host. The majority of antibodies targeting spike RBDs bear neutralization function which in part determines the spread of CoV viruses. The positive sample in LFD assays displayed decent responses to SARS-CoV-2 and reduced binding to Omicron (**Figures 2D**–**F**), aligning with recent reports (8). Carreno et al. found that the eukaryotic expressed monomeric RBD of Omicron reduced binding activity to convalescent and vaccine (mRNA-1273 and BNT162b2) serum with a more than 1.5-fold drop (8). Cameroni et al. demonstrated that most receptor-binding motif (RBM)-directed monoclonal antibodies (mAbs) lost *in vitro* neutralizing activity against Omicron (8). High-throughput yeast display screening assays from Cao et al. revealed that over 85% of the RBD-neutralizing antibodies were escaped by Omicron (8). Neutralizing assays using authentic and pseudotype viruses indicated that the Omicron variant showed lower neutralizing sensitivity than other SARS-CoV-2 variants to convalescent and vaccine (mRNA1273, BNT162b2, BBIBP-CorV, and ZF2001) serum (8). All these data suggest that omicron can penetrate the vaccine-induced immune barrier, which explained at least in part the quick spread of Omicron. One thing that needs to be emphasized is that even in the vaccinated hosts who are negative in Omicron RBD-specific antibodies, the preexisting SARS-CoV-2-specific memory B cells and T cells can provide protection in the following Omicron infection, although they may contribute less to inhibit the entry of Omicron into host cells (8). This is possibly the reason why Omicron spreads rapidly but does not induce more severe symptoms.

Collectively, our study demonstrates that Omicron RBD displays a lower reactivity to prototype SARS-CoV-2 vaccine-immunized human sera as compared to homogeneous SARS-CoV-2 RBD, implying the insufficient protection of the prototype SARS-CoV-2 vaccine against the Omicron variant (**Figure 2H**). The booster of the prototype SARS-CoV-2 vaccine enhances the level of antibodies against both the SARS-CoV-2 prototype and the Omicron variant, which can help defend against the COVID-19 pandemic. Omicron RBD reactivity to SARS-CoV-2 vaccine-immunized human sera requires to be assessed on a large scale.

## Data availability statement

The original contributions presented in the study are included in the article/**supplementary material**. Further inquiries can be directed to the corresponding authors.

## Ethics statement

The animal study was reviewed and approved by the Jiangxi Agricultural University Ethics Committee with the protocol number JXAU20220007.

## Author contributions

ML, SW, and QW contributed equally to this work. TW, ML, SW, and QW performed the majority of experiments, interpreted the data, and prepared the figures and tables. ZY, LZ, and FT performed the protein expression and denaturation. YY, QZ, MJ, and LY collected the samples and prepared the relevant information. XX, HL, and LK conducted the data analysis. XW and YL polished the draft. YW, SL, and LK developed the research plan and experimental strategy, analyzed the data, and wrote the paper. All authors contributed to the article and approved the submitted version.

## Funding

This study was funded by research grants from the National Natural Science Foundation of China (grants 31460667, 31860038, and 31960699), Jiangxi Province (grants 20161BBF60084, GJJ180239, and GJJ200405), and Jiangxi Agriculture University (9232307726).

## Acknowledgments

The authors would like to thank Dr. Feng Cong (Guangdong Laboratory Animals Monitoring Institute) for kindly providing a recombinant plasmid-carrying prototype SARS-CoV-2 spike gene (GenBank: YP_009724390), as well as Ms. Yanni Zhang (Jiangxi Province Center for Disease Control and Prevention) and Dr. Xianfeng Zhou (Nanchang City Center for Disease Control and Prevention) for technical support.

## Conflict of interest

The authors declare that the research was conducted in the absence of any commercial or financial relationships that could be construed as a potential conflict of interest.

## Publisher’s note

All claims expressed in this article are solely those of the authors and do not necessarily represent those of their affiliated organizations, or those of the publisher, the editors and the reviewers. Any product that may be evaluated in this article, or claim that may be made by its manufacturer, is not guaranteed or endorsed by the publisher.

## Supplementary material

Supplementary Figure 1 Examination of anti-SARS-CoV-2 spike protein antibodies in human immunized sera using LFD assays. **(A)** The intensity of LFD bands in was quantified using Image J. (B-C) Adult sera collected at month 1, 5, or 6 post the second round of vaccination were examined with SARS-CoV-2 Antibody Detection Kit from Vazyme **(B)** or Innovita **(C)**. **(D)** Adult sera collected at month 1 or day 5 post the third round of vaccination were examined with SARS-CoV-2 Antibody Detection Kit from Wondfo. **(E-F)** Children’s sera collected at different time points after vaccination and unimmunized children’s sera were examined with SARS-CoV-2 Antibody Detection Kit from Wondfo. Further details were provided in **Supplementary Table 1**.

Supplementary Figure 2 Identification of pre-coated recombinant RBDs by anti-His tag antibody. Recombinant RBDs were examined by ELISA assays with anti-His tag antibody, vaccine serum (participant ID: 31-1), and unimmunized sera (UC-sera, participant ID: 62-1), or PBS.
