# Supplementary material for: Reduced binding activity of vaccine serum to omicron receptor-binding domain
Source: Front Immunol. 2022 Jul 28;13:960195. doi: 10.3389/fimmu.2022.960195 (PMC9369000; doi:10.3389/fimmu.2022.960195)

Supplementary Fig.1

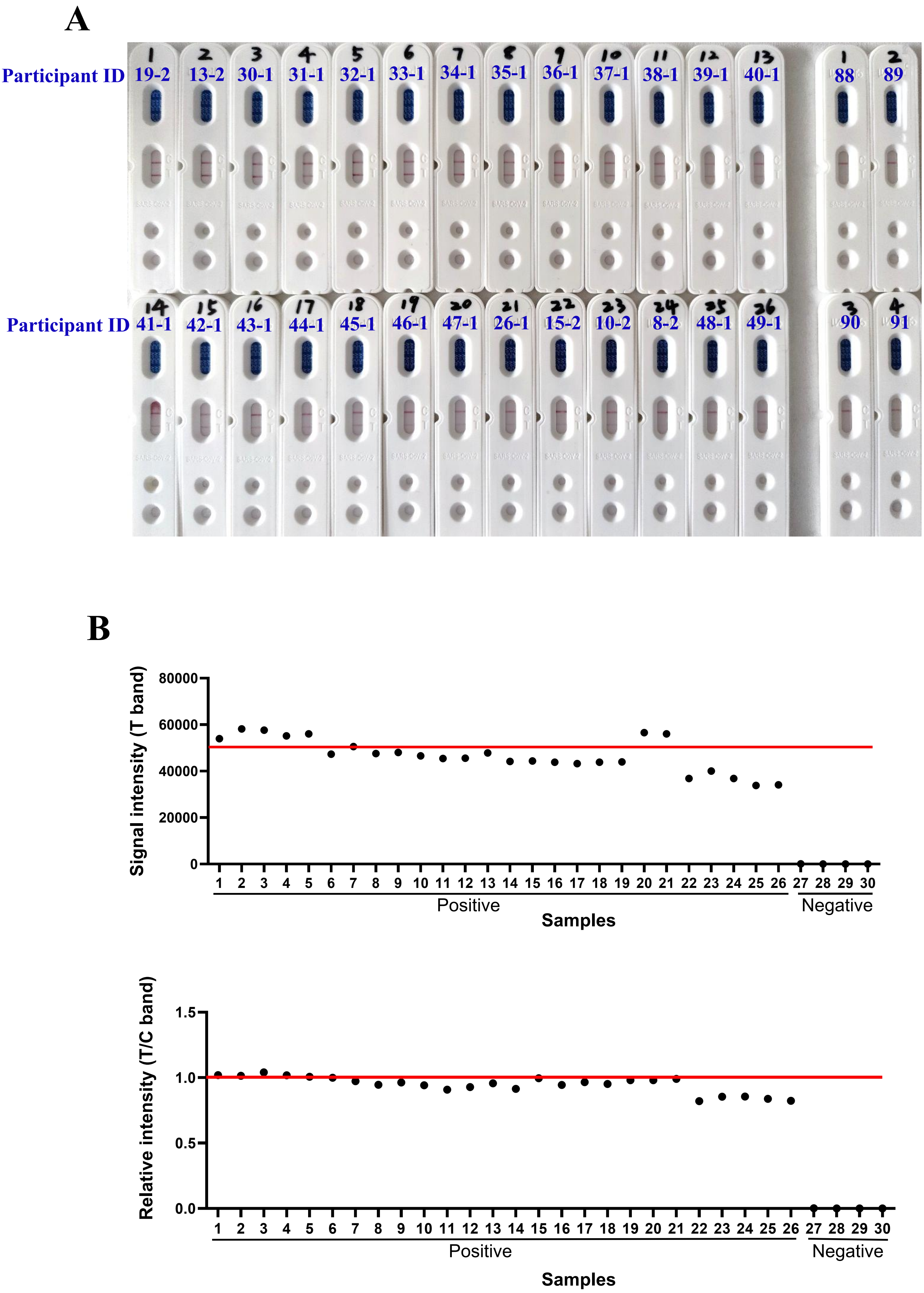

Supplementary Fig.1

C

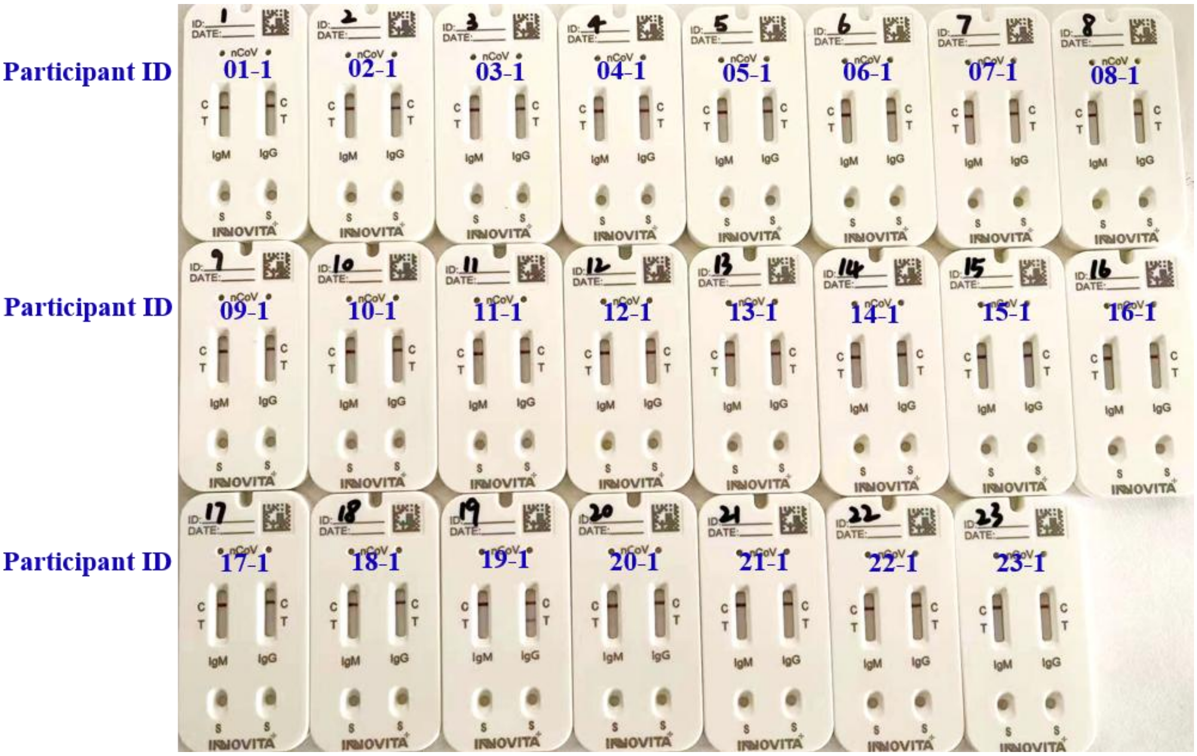

D

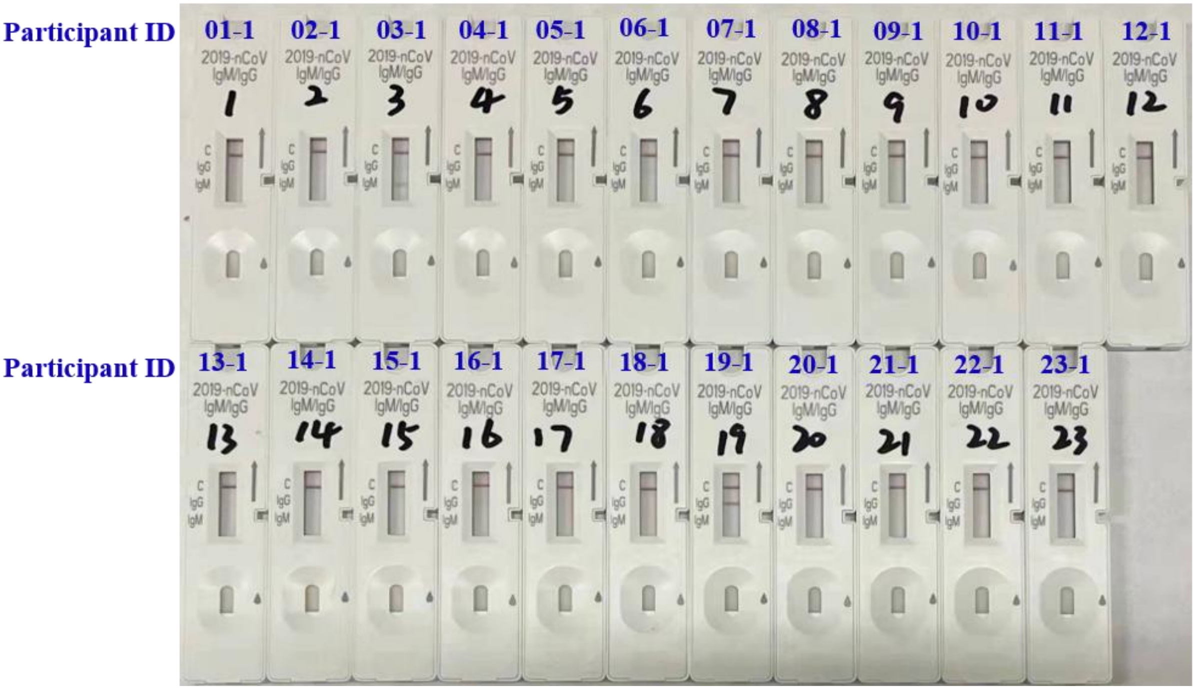

Supplementary Fig.1

E

Participant ID

Participant ID

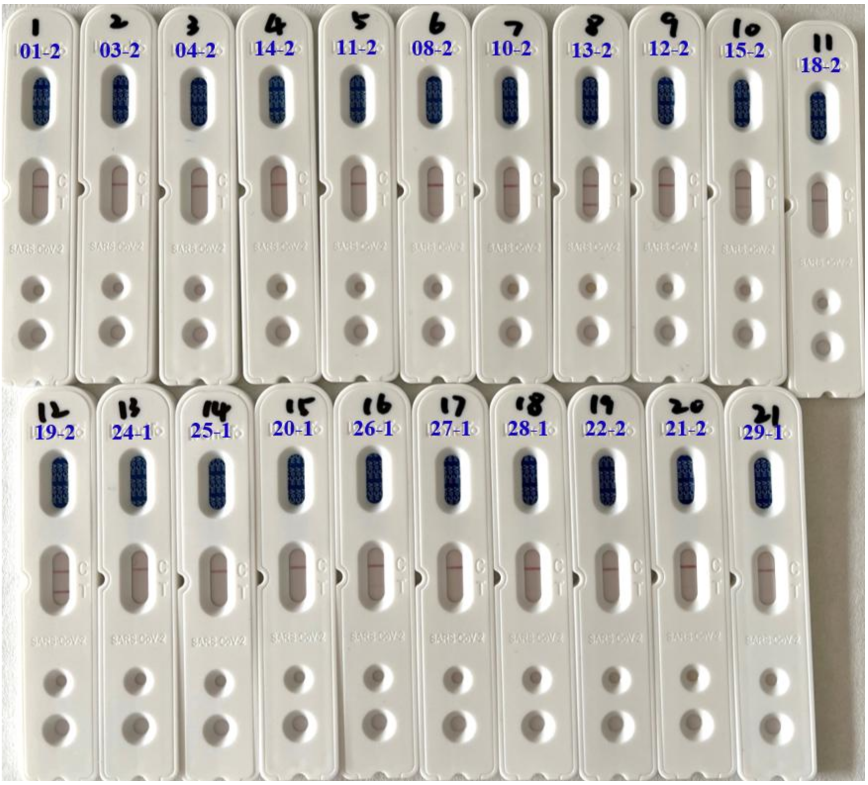

F

Participant ID

Participant ID

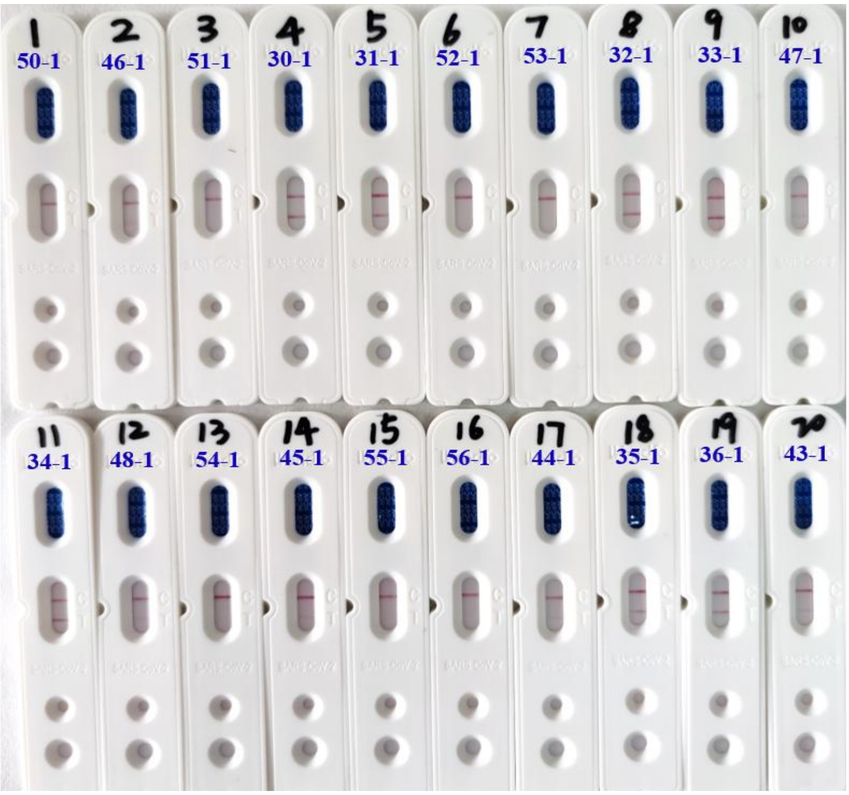

Supplementary Fig.1

G

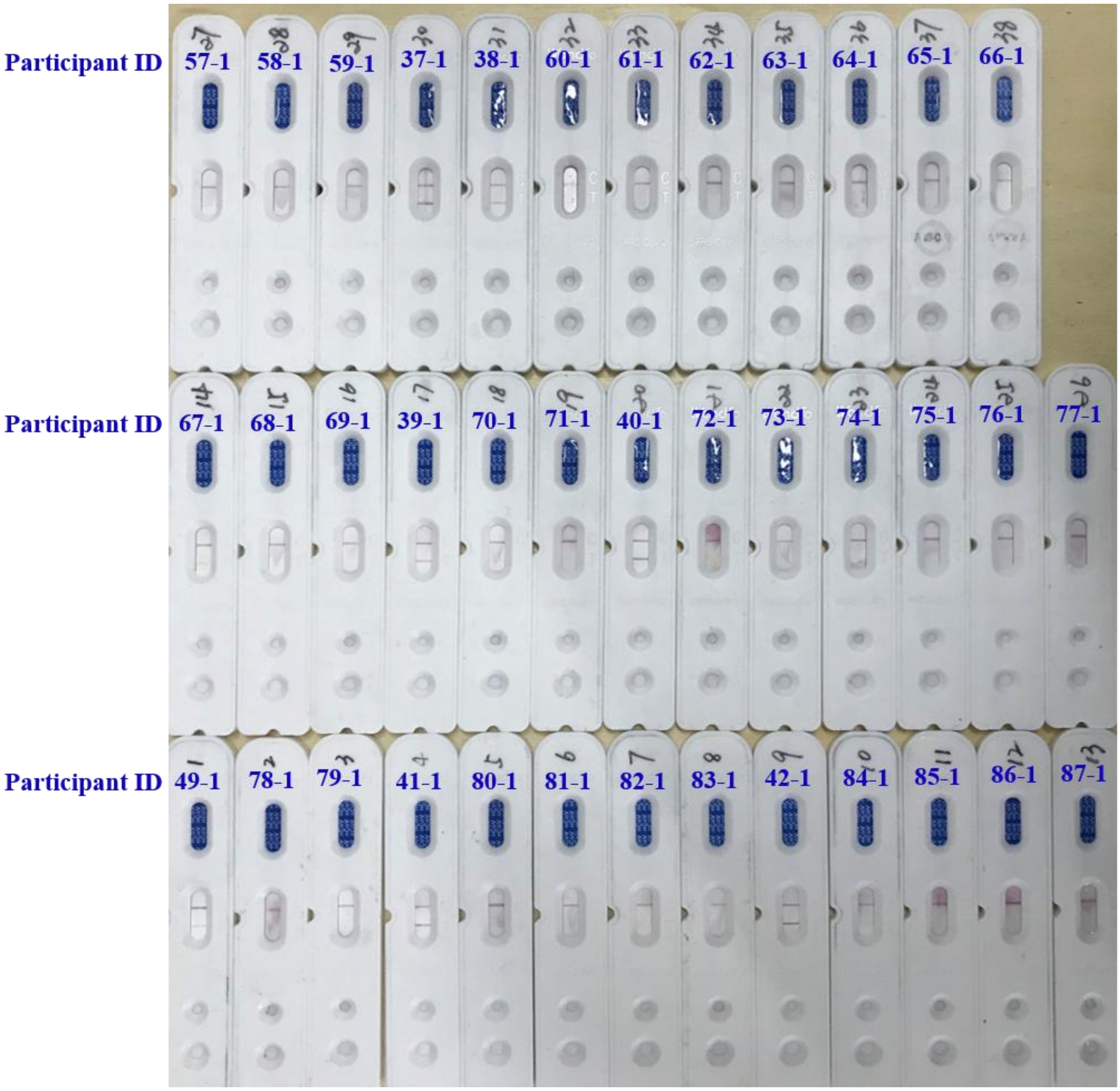

Supplementary Fig.2

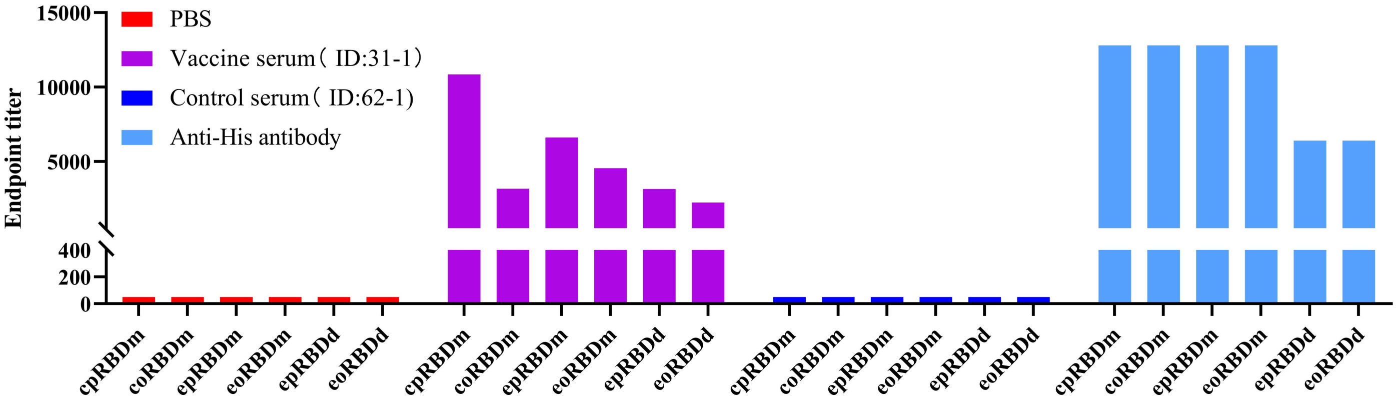

Supplement: Supplementary Figure 1 — Examination of anti-SARS-CoV-2 spike protein antibodies in human immunized sera using LFD assays. (A) The intensity of LFD bands in was quantified using Image J. (B-C) Adult sera collected at month 1, 5, or 6 post the second round of vaccination were examined with SARS-CoV-2 Antibody Detection Kit from Vazyme (B) or Innovita (C). (D) Adult sera collected at month 1 or day 5 post the third round of vaccination were examined with SARS-CoV-2 Antibody Detection Kit from Wondfo. (E-F) Children’s sera collected at different time points after vaccination and unimmunized children’s sera were examined with SARS-CoV-2 Antibody Detection Kit from Wondfo. Further details were provided in Supplementary Table 1. [file Image_1.pdf]
