# Supplementary material for: Reduced binding activity of vaccine serum to omicron receptor-binding domain
Source: Front Immunol. 2022 Jul 28;13:960195. doi: 10.3389/fimmu.2022.960195 (PMC9369000; doi:10.3389/fimmu.2022.960195)
Supplement: Supplementary file 2 [file Table_1.pdf]

Supplementary table 1 Detailed description of samples used in this study

Participant ID	Age	Sex	Ancestry	Time points included in this study	SARS-CoV-2 infection prior to vaccination	Vaccine Type
01-1/-2	49	Male	Chinese	First-Boost (6 months), Second-Boost (5 days)	No	Sinovac, Sinopharm
02-1	44	Female	Chinese	First-Boost (5 months)	No	Sinopharm, Sinovac
03-1/-2	34	Female	Chinese	First-Boost (6 months), Second-Boost (5 days)	No	Sinovac, Sinopharm
04-1/-2	33	Female	Chinese	First-Boost (6 months), Second-Boost (5 days)	No	Sinovac, Sinopharm
05-1	30	Male	Chinese	First-Boost (6 months)	No	Sinovac, Sinopharm
06-1	29	Female	Chinese	First-Boost (5 months)	No	Sinovac, Sinopharm
07-1	28	Male	Chinese	First-Boost (5 months)	No	Sinovac, Sinopharm
08-1/-2	25	Male	Chinese	First-Boost (6 months), Second-Boost (6 days)	No	Sinovac, Sinopharm
09-1	25	Female	Chinese	First-Boost (5 months)	No	Sinovac, Sinopharm
10-1/-2	25	Female	Chinese	First-Boost (6 months), Second-Boost (6 days)	No	Sinovac, Sinopharm
11-1/-2	25	Male	Chinese	First-Boost (6 months), Second-Boost (6 days)	No	Sinovac, Sinopharm
12-1/-2	26	Female	Chinese	First-Boost (6 months), Second-Boost (6 days)	No	Sinovac, Sinopharm
13-1/-2	24	Male	Chinese	First-Boost (6 months), Second-Boost (6 days)	No	Sinovac, Sinopharm
14-1/-2	31	Female	Chinese	First-Boost (6 months), Second-Boost (5 days)	No	Sinovac, Sinopharm
15-1/-2	23	Female	Chinese	First-Boost (6 months), Second-Boost (6 days)	No	Sinovac, Sinopharm
16-1	24	male	Chinese	First-Boost (5 months)	No	Sinovac, Sinopharm
17-1	24	Female	Chinese	Second-Boost (5 months)	No	Zhifei Longcom
18-1/-2	24	Female	Chinese	First-Boost (6 months), Second-Boost (6 days)	No	Sinopharm, Sinovac
19-1/-2	23	Male	Chinese	First-Boost (6 months), Second boost (1 month)	No	Sinovac, Sinopharm
20-1/-2	22	Female	Chinese	First-Boost (6 months), Second-Boost (5 days)	No	Sinovac, Sinopharm
21-1/-2	20	Female	Chinese	First-Boost (6 months), Second-Boost (5 days)	No	Sinovac, Sinopharm

22-1/-2	20	Male	Chinese	First-Boost (6 months), Second-Boost (5 days)	No	Sinovac, Sinopharm
23-1	24	Female	Chinese	First-Boost (5 months)	No	Sinovac, Sinopharm
24-1	19	Female	Chinese	Second-Boost (5 days)	No	Sinovac, Sinopharm
25-1	21	Male	Chinese	Second-Boost (5 days)	No	Sinovac, Sinopharm
26-1	21	Female	Chinese	Second-Boost (5 days)	No	Sinovac, Sinopharm
27-1	19	Male	Chinese	Second-Boost (5 days)	No	Sinovac, Sinopharm
28-1	21	Male	Chinese	Second-Boost (5 days)	No	Sinovac, Sinopharm
29-1	19	Female	Chinese	Second-Boost (5 days)	No	Sinovac, Sinopharm
30-1	8	Male	Chinese	First-Boost (1 month)	No	Sinovac
31-1	10	Male	Chinese	First-Boost (1 month)	No	Sinovac
32-1	12.5	Male	Chinese	First-Boost (12 days)	No	Sinovac
33-1	7	Male	Chinese	First-Boost (20 days)	No	Sinovac
34-1	7	Female	Chinese	First-Boost (7 days)	No	Sinopharm
35-1	12	Female	Chinese	First-Boost (7 days)	No	Sinopharm
36-1	8.5	Male	Chinese	Second-Boost (7 days)	No	Sinopharm
37-1	7	Male	Chinese	First-Boost (7 days)	No	Sinopharm
38-1	8.5	Female	Chinese	First-Boost (22 days)	No	Sinopharm
39-1	6.5	Female	Chinese	First-Boost (20 days)	No	Sinovac, Sinopharm
40-1	4.5	Female	Chinese	First-Boost (1 month)	No	Sinovac, Sinopharm
41-1	8.5	Male	Chinese	First-Boost (7 days)	No	Sinovac, Sinopharm
42-1	5.5	Male	Chinese	First-Boost (21 days)	No	Sinopharm, Sinovac
43-1	6.5	Female	Chinese	First-Boost (15 days)	No	Sinovac
44-1	9.5	Female	Chinese	Second-Boost (7 days)	No	Sinopharm, Sinovac
45-1	5.5	Male	Chinese	First-Boost (21 days)	No	Sinovac
46-1	10	Male	Chinese	First-Boost (7 days)	No	Sinovac

47-1	11	Male	Chinese	First-Boost (15 days)	No	Sinovac
48-1	10.5	Female	Chinese	First-Boost (7 days)	No	Sinopharm
49-1	5	Male	Chinese	First-Boost (7 days)	No	Sinopharm
50-1	2	Female	Chinese	No	No	No
51-1	2	Male	Chinese	No	No	No
52-1	4	Male	Chinese	Primary immunization (10 days)	No	Sinovac
53-1	9	Male	Chinese	First-Boost (3 months)	No	Sinopharm, Sinovac
54-1	3	Female	Chinese	No	No	No
55-1	6.5	Female	Chinese	Primary immunization (10 days)	No	Sinovac
56-1	6.5	Male	Chinese	First-Boost (3 months)	No	Sinovac, Sinopharm
57-1	1	Male	Chinese	No	No	No
58-1	6	Male	Chinese	Primary immunization (7 days)	No	Sinovac
59-1	2.5	Female	Chinese	No	No	No
60-1	4.5	Female	Chinese	First-Boost (4 months)	No	Sinopharm
61-1	7.5	Female	Chinese	First-Boost (2 months)	No	Sinovac, Sinopharm
62-1	2.5	Female	Chinese	No	No	No
63-1	7	Male	Chinese	Second-Boost (5 days)	No	Sinovac, Sinopharm
64-1	5.5	Female	Chinese	First-Boost (1 month)	No	Sinopharm
65-1	8	Male	Chinese	First-Boost (5 months)	No	Sinovac, Sinopharm
66-1	3	Female	Chinese	Primary immunization (7 days)	No	Sinopharm
67-1	3	Male	Chinese	Primary immunization (7 days)	No	Sinovac
68-1	6	Male	Chinese	First-Boost (3 months)	No	Sinovac, Sinopharm
69-1	7	Female	Chinese	First-Boost (4 months)	No	Sinopharm
70-1	5.5	Male	Chinese	First-Boost (3 months)	No	Sinovac, Sinopharm
71-1	6.5	Male	Chinese	First-Boost (4 months)	No	Sinovac, Sinopharm

72-1	8	Male	Chinese	First-Boost (3 months)	No	Sinopharm
73-1	5.5	Female	Chinese	Primary immunization (10 days)	No	Sinovac
74-1	3	Female	Chinese	Primary immunization (7 days)	No	Sinovac
75-1	9	Male	Chinese	First-Boost (2 months)	No	Sinovac, Sinopharm
76-1	0.5	Male	Chinese	No	No	No
77-1	6	Male	Chinese	First-Boost (3 months)	No	Sinovac, Sinopharm
78-1	3.5	Female	Chinese	First-Boost (20 days)	No	Sinopharm
79-1	3.5	Female	Chinese	First-Boost (1 month)	No	Sinovac, Sinopharm
80-1	9	Male	Chinese	First-Boost (5 months)	No	Sinovac, Sinopharm
81-1	5	Male	Chinese	First-Boost (20 days)	No	Sinopharm
82-1	1.5	Male	Chinese	No	No	No
83-1	10	Male	Chinese	First-Boost (4 months)	No	Sinovac, Sinopharm
84-1	3	Male	Chinese	Primary immunization (10 days)	No	Sinopharm
85-1	2.5	Male	Chinese	No	No	No
86-1	6	Male	Chinese	First-Boost (4 months)	No	Sinopharm
87-1	8	Female	Chinese	First-Boost (5 months)	No	Sinovac, Sinopharm
88-1	0.75	Male	Chinese	No	No	No
89-1	0.75	Female	Chinese	No	No	No
90-1	4 days	Female	Chinese	No	No	No
91-1	15 days	Male	Chinese	No	No	No

For Participant ID, the first digit represents the sequential number of participants, which is followed by times of sample collection separated with slash. The positive serum samples identified by colloidal gold were highlighted with red while the negative sera of children no more than 3 years old were highlighted with yellow. For CoronaVac vaccine, recommended 3μg dosage was used (Han et al., 2021; Wu et al., 2021). For BBIBP-CorV vaccine, recommended 4μg dosage was used(Wang et al., 2022). For ZF2001 vaccine, recommended 25μg dosage for adults was used(Dai et al., 2020; Yang et al., 2021).

Supplementary table 2： Positive situation of vaccine sera in LFD assay by the vaccine, age, sex, and post-immunization time

Group		Post-immunization Time (Days)		
		<7	7-30	>30
Vaccine Type	Sinovac + Sinopharm	5/21(Second-Boost)	4/5(First-Boost),<br>1/1(Second-Boost)	0/34(First-Boost),<br>1/1(Second-Boost)
	Sinopharm	None	6/8(First-Boost),<br>1/1(Second-Boost),<br>0/2(Primary immunization)	0/5(First-Boost)
	Sinovac	None	8/8(First-Boost),<br>0/6(Primary immunization)	None
	Zhifei Longcom	None	None	0/1(Second-Boost)
Age (Year)	3-7	0/1(Second-Boost)	9/12(First-Boost),<br>0/8(Primary immunization)	0/9(First-Boost)
	8-13	None	9/9(First-Boost),<br>2/2(Second-Boost)	0/8(First-Boost)
	18-30	5/16(Second-Boost)	None	0/17(First-Boost),<br>1/2(Second-Boost)
	31-50	0/4(Second-Boost)	None	0/5(First-Boost)
Sex	male	2/9(Second-Boost)	11/12(First-Boost),<br>1/1(Second-Boost),<br>0/4(Primary immunization)	0/20(First-Boost),<br>1/1(Second-Boost)
	female	3/12(Second-Boost)	7/9(First-Boost),<br>1/1(Second-Boost)<br>0/4(Primary immunization)	0/19(First-Boost),<br>0/1(Second-Boost)

References:

Dai, L., Zheng, T., Xu, K., Han, Y., Xu, L., Huang, E., et al. (2020). A Universal Design of Betacoronavirus Vaccines against COVID-19, MERS, and SARS. *Cell* 182(3)**,** 722-733 e711. doi: 10.1016/j.cell.2020.06.035.

Han, B., Song, Y., Li, C., Yang, W., Ma, Q., Jiang, Z., et al. (2021). Safety, tolerability, and immunogenicity of an inactivated SARS-CoV-2 vaccine (CoronaVac) in healthy children and adolescents: a double-blind, randomised, controlled, phase 1/2 clinical trial. *Lancet Infect Dis* 21(12)**,** 1645-1653. doi: 10.1016/S1473-3099(21)00319-4.

Wang, C., Chen, L.Y., Lu, Q.B., and Cui, F. (2022). Vaccination with the Inactivated Vaccine (Sinopharm BBIBP-CorV) Ensures Protection against SARS-CoV-2 Related Disease. *Vaccines (Basel)* 10(6). doi: 10.3390/vaccines10060920.

Wu, Z., Hu, Y., Xu, M., Chen, Z., Yang, W., Jiang, Z., et al. (2021). Safety, tolerability, and immunogenicity of an inactivated SARS-CoV-2 vaccine (CoronaVac) in healthy adults aged 60 years and older: a randomised, double-blind, placebo-controlled, phase 1/2 clinical trial. *Lancet Infect Dis* 21(6)**,** 803-812. doi: 10.1016/S1473-3099(20)30987-7.

Yang, S., Li, Y., Dai, L., Wang, J., He, P., Li, C., et al. (2021). Safety and immunogenicity of a recombinant tandem-repeat dimeric RBD-based protein subunit vaccine (ZF2001) against COVID-19 in adults: two randomised, double-blind, placebo-controlled, phase 1 and 2 trials. *Lancet Infect Dis* 21(8)**,** 1107-1119. doi: 10.1016/S1473-3099(21)00127-4.
